# Supplementary material for: The Antimicrobial Potential and Aquaculture Wastewater Treatment Ability of Penaeidins 3a Transgenic Duckweed
Source: Plants (Basel). 2023 Apr 20;12(8):1715. doi: 10.3390/plants12081715 (PMC10144588; doi:10.3390/plants12081715)
Supplement: Supplementary file 1 [file plants-12-01715-s001.zip › plants-2269299-supplementary.pdf]

Supplementary Materials:

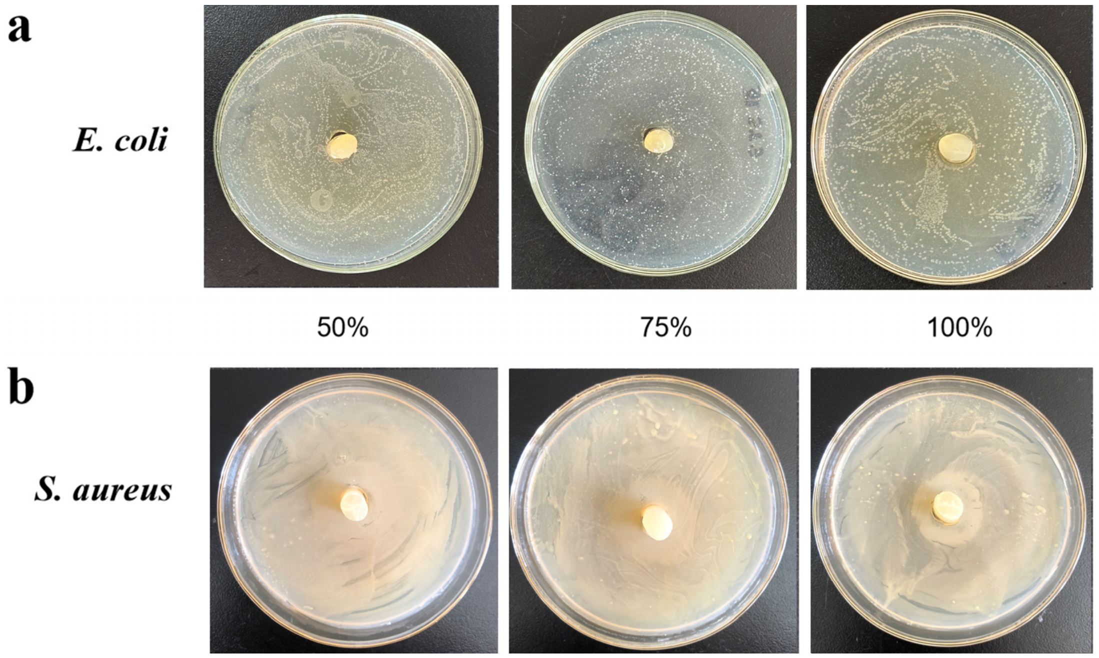

Figure S1. Zone of inhibition of various bacteria by WT duckweed extracting solution. (**a**) Inhibition zone of different concentrations WT duckweed extracting solution on *Escherichia coli*. (**b**) Inhibition zone of different concentrations WT duckweed extracting solution on *Staphylococcus aureus.*

Table S1 Gene expression of Penaeidin in duckweed

Lines	Gene-id	Description	readcount
Pen3a_D	Cluster-6169.0	Penaeidin-3 OS=*Litopenaeus vannamei*	1.84520081740231
Pen3a_E	Cluster-6169.0	Penaeidin-3 OS=*Litopenaeus vannamei*	5.418213813
Pen3a_H	Cluster-6169.0	Penaeidin-3 OS=*Litopenaeus vannamei*	5.67877520684982
Pen3a_J	Cluster-6169.0	Penaeidin-3 OS=*Litopenaeus vannamei*	4.75452543036578
Pen3a_K	Cluster-6169.0	Penaeidin-3 OS=*Litopenaeus vannamei*	18.3589398900158
WT	Cluster-6169.0	Penaeidin-3 OS=*Litopenaeus vannamei*	0
